# Mechanisms and immunogenicity of nsPEF-induced cell death in B16F10 melanoma tumors

**DOI:** 10.1038/s41598-018-36527-5

**Published:** 2019-01-23

**Authors:** Alessandra Rossi, Olga N. Pakhomova, Andrei G. Pakhomov, Samantha Weygandt, Anna A. Bulysheva, Len E. Murray, Peter A. Mollica, Claudia Muratori

**Affiliations:** 1 Old Dominion University, Frank Reidy Research Center for Bioelectrics, Norfolk, VA 23508 USA; 2 SoBran Inc., 700 West Olney Road, Norfolk, VA 23507 USA; 3 Department of Medical Diagnostics and Translational Sciences, Old Dominion University, Norfolk, VA 23508 USA

## Abstract

Accumulating data indicates that some cancer treatments can restore anticancer immunosurveillance through the induction of tumor immunogenic cell death (ICD). Nanosecond pulsed electric fields (nsPEF) have been shown to efficiently ablate melanoma tumors. In this study we investigated the mechanisms and immunogenicity of nsPEF-induced cell death in B16F10 melanoma tumors. Our data show that *in vitro* nsPEF (20–200, 200-ns pulses, 7 kV/cm, 2 Hz) caused a rapid dose-dependent cell death which was not accompanied by caspase activation or PARP cleavage. The lack of nsPEF-induced apoptosis was confirmed *in vivo* in B16F10 tumors. NsPEF also failed to trigger ICD-linked responses such as necroptosis and autophagy. Our results point at necrosis as the primary mechanism of cell death induced by nsPEF in B16F10 cells. We finally compared the antitumor immunity in animals treated with nsPEF (750, 200-ns, 25 kV/cm, 2 Hz) with animals were tumors were surgically removed. Compared to the naïve group where all animals developed tumors, nsPEF and surgery protected 33% (6/18) and 28.6% (4/14) of the animals, respectively. Our data suggest that, under our experimental conditions, the local ablation by nsPEF restored but did not boost the natural antitumor immunity which stays dormant in the tumor-bearing host.

## Introduction

The term immunogenic cell death (ICD) indicates a cell death modality that stimulates an adaptive immune response against dead-cell associated antigens. The immune-stimulating capacity of ICD depends on the regulated emission of damage-associated molecular patterns (DAMPs), such as the endoplasmic reticulum protein calreticulin (CRT), ATP and the chromatin- binding protein high mobility group B1 (HMGB1)^1^. Collectively, these ICD-associated DAMPs recruit antigen presenting cells to the tumor site enhancing their ability to engulf, process and present tumor-derived antigens to T cells, thus favoring the induction of a tumor specific adaptive immunity^1^.

For years, it was generally accepted that DAMPs released during necrosis can lead to a local inflammation and generate immune responses. However, many attempts to generate successful immune response using necrotic cells failed^2,3^. On the other hand at least some death stimuli triggering apoptosis, a cell death mode generally considered non-immunogenic, were able to mount successful adaptive immunity. For instance when doxorubicin-treated apoptotic colorectal cancer CT-26 cells were injected subcutaneously into BALB/c mice, they induced an immune response that protected the mice against a subsequent challenge with live cells of the same type^4^. These results revealed, for the first time, that a caspase dependent modality of apoptosis could stimulate an anticancer immunosurveillance. Recently cells undergoing necroptosis, a regulated form of necrosis, were shown to exhibit all biochemical features of ICD^5^. Hence different regulated cell death modalities (apoptosis and necroptosis) can contribute to ICD.

One common feature of all ICD stimuli so far identified is their capacity to induce *premortem* stress responses such as reactive oxygen species (ROS)-based endoplasmic reticulum (ER) stress and autophagy^6^. These stress responses lead to the release and exposure of DAMPs required for ICD. Therefore, it is not only the cell death subroutine but a combination of both stress response and cell death that yield ICD. For example, translocation of CRT to the outer leaflet of the plasma membrane requires three signaling modules: ER stress, apoptosis and a terminal translocation module which expose CRT on the cell plasma membrane. On the other hand, active ATP release involves a two-step mechanism which involves the activation of the autophagic machinery along with the execution of apoptosis^7^.

From the above discussion it is clear that there is a close association between cell death pathways and the emission and trafficking of DAMPs; such that in certain cases, the trafficking of DAMPs itself might be regulated by signaling pathways that execute cell death.

Nanosecond pulsed electric fields (nsPEF) are emerging as a new promising modality for tumor and tissue ablation. In addition to their high ablation efficiency, several studies reported that tumor ablation using nsPEF can induce an antitumor immune response^8–13^.

The best known primary effect of nsPEF is the permeabilization of membranes including the plasma membrane, mitochondria, and endoplasmic reticulum^14–19^. Immediate effects of membrane permeabilization include calcium mobilization^17–21^, cell swelling, blebbing, and disassembly of actin structures^22–24^. Cell damage by nsPEF was found to trigger stress response pathways such as autophagy^25^ and, when the damage exceeded repairable limits, necrosis and apoptosis^15,26–28^. Although electropermeabilization is a well-established cause for nsPEF bioeffects, it is not necessarily the only mechanism. Indeed, nsPEF were found to generate ROS production^29,30^. Along with membrane permeabilization, the anti-oxidant defense and ROS formation may be among the factors that determine the cytotoxic effect and the efficiency of tumor ablation by nsPEF.

The exact mechanisms responsible for nsPEF cytotoxicity have been the subject of numerous studies^15,26–28,31–37^. Early studies reported apoptosis as the prevailing or even the sole mode of cell death after nsPEF^31,38,39^. Indeed, various cell types exposed to lethal nsPEF doses show hallmarks of apoptosis such as caspase activation, DNA fragmentation, cytochrome *C* release in the cytoplasm, and poly-ADP ribose polymerase (PARP) cleavage^15,27,31,40,41^.

Concurrently, several groups including ours reported that cells treated with nsPEF swell and die of primary necrosis within a few hours after the treatment^26,27,40,42–45^. Necrosis is caused by the loss of membrane integrity, and colloid-osmotic imbalance which leads to cell swelling and membrane rupture^26^.

The aforementioned studies used rather different tumor models and pulse treatments (as defined by the pulse width, number of pulses, amplitude and pulse repetition rate) suggesting that the balance between necrosis and apoptosis could be influenced by these factors. Indeed, the dependence of the cytotoxic effect upon exposure parameters remains poorly understood.

Despite this incomplete knowledge, nsPEF have already been employed to ablate many different types of tumors^11,42,46–48^. The results have been successful, tumors are eliminated in one treatment without recurrence and with minimal side effects.

Recently several studies reported that nsPEF ablation inhibits secondary tumors growth^8–13^. It has been hypothesized that the mechanism responsible for this inhibition involves an adaptive immune response stimulated by the cell death modality triggered by the nsPEF treatment. To support this hypothesis, Nuccitelli *et al*. recently reported apoptotic cell death accompanied by CTR exposure, ATP and HMGB1 release in response to 100-ns pulses in three different tumor cell types (MCA 205 murine fibrosarcoma, Jurkat E6-1 human T-cell leukemia and McA-RH777 rat hepatocarcinoma)^13^.

Given that the pulse parameters can affect the cell death modality triggered by nsPEF, and that specific cell death pathways regulate the immunogenicity of the cell death, it should come as no surprise that protocols that are equally efficient at killing tumors *in vivo* may not elicit the same antitumor immune response. For instance the lack of caspase activation in response to nsPEF may impair DAMPs emission and therefore reduce the immunogenicity of the treatment. Indeed the capacity to initiate an effective antitumor immunity after nsPEF ablation is highly variable. While two studies using orthotopic models of rat hepatocellular carcinoma and mouse mammary carcinoma showed that nsPEF protected 100% of the treated animals against tumor challenge^8,9^, other groups reported a long-lasting antitumor immunity in only 25% of the treated animals^10^.

The aim of the present study was to investigate the mechanisms and immunogenicity of nsPEF-induced cell death. We chose a tumor model which has been extensively studied for nsPEF ablation, namely the murine melanoma B16F10 model. Tumors of about 40–50 mm^3^ were treated with 200-ns pulses (500–750, 25 kV/cm, 2 Hz) and, 7 weeks post treatment, animals that experienced complete tumor remission were challenged with the same tumor cell type. At 50 days post challenge, 33% of the animals were protected from secondary tumor growth. We investigated whether nsPEF triggered apoptosis, necroptosis and autophagy. None of these pathways involved in the active emission of DAMPs were activated suggesting that the nsPEF treatment triggered primarily necrosis. We finally compared the antitumor immunity in animals treated with nsPEF with animals were tumors were surgically removed. After challenge, both experimental groups showed similar percentage of tumor free animals. These data suggest that the local cell death triggered by nsPEF restore but did not boost the natural occurring antitumor immunity which stays dormant co-existing with the progressing tumor.

## Results

### NsPEF ablated primary B16F10 melanomas and protected 33% of the animals from tumor challenge

To study the correlation between nsPEF-induced antitumor immunity and the specific cell death pathways triggered by the pulse treatment, we used the C57BL/6 syngeneic B16F10 melanoma cell line. This model has been extensively used to study nsPEF ablation^42,49,50^ but the protective antitumor response triggered by the pulse treatment has not yet been investigated. In preliminary experiments we investigated the antitumor efficacy of 200-ns pulses over a range of pulse numbers (300–1000 pulses, 25 kV/cm, 2 Hz). At 24 h post treatment, both 500 and 750 pulses cause complete tumor destruction, and therefore these nsPEF doses were selected to study the antitumor immune response (data not shown).

**Figure 1:**
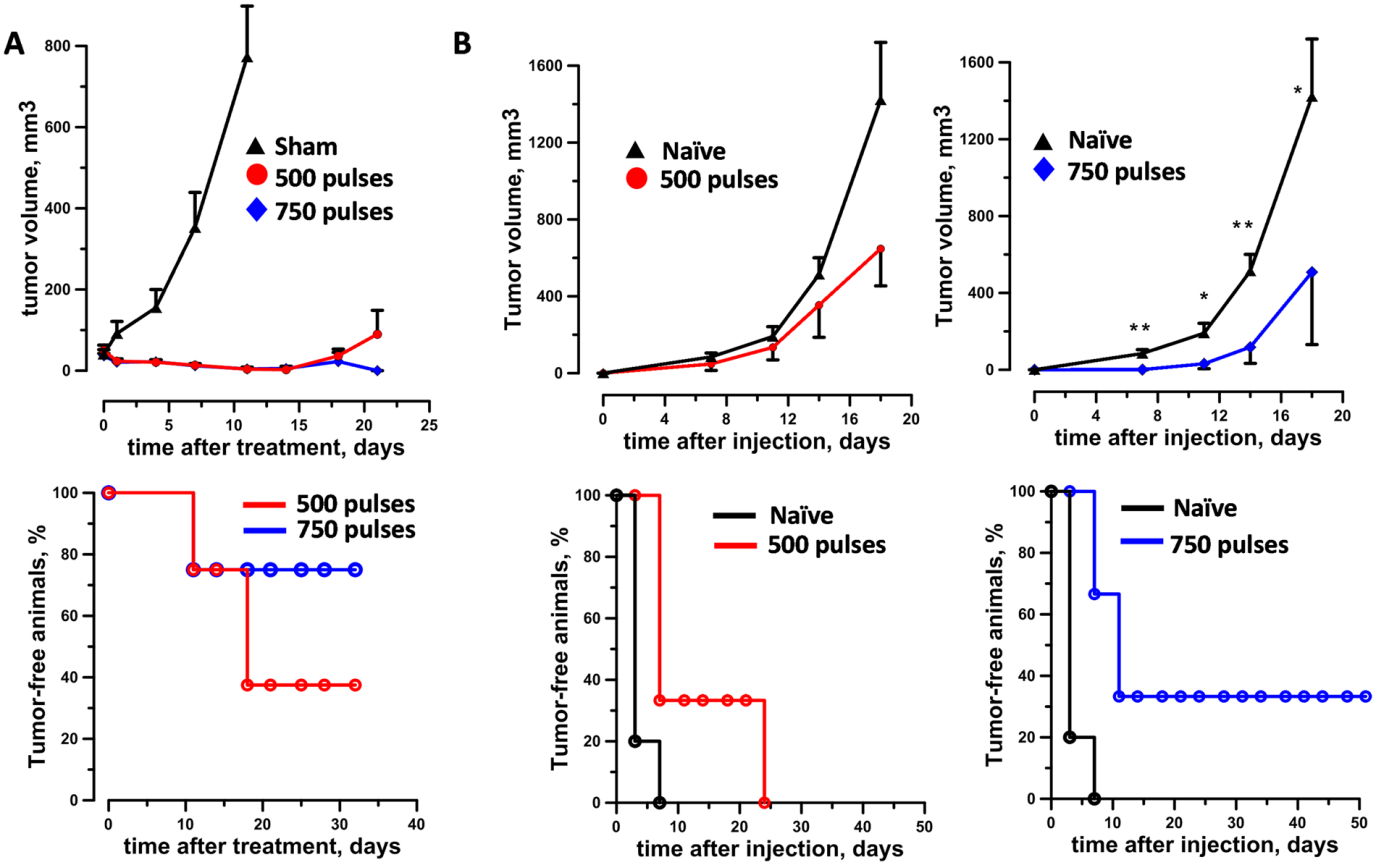
Ablation and protective antitumor immune response induced by nsPEF in B16F10 melanoma model. Mice bearing 40–50 mm^3^ B16F10 tumors were treated with either 500 or 750, 200-ns pulses (25 kV/cm, 2 Hz) or left untreated (sham control). At 7 weeks post nsPEF, animals that experienced complete tumor remission were challenged with tumor cells and monitored for the appearance of palpable tumors. An age matched naïve group was used as a control for tumor growth. Panels A and B show the tumor growth curves (top graphs) and % of tumor free animals (bottom graphs) after nsPEF (**A**) and tumor challenge (**B**), respectively. Mean ± s.e., n = 8 for sham, 500 and 750 pulses (**A**). Mean ± s.e., n = 10 and n = 3 for naïve and 500 pulses, respectively (**B**, left graphs). Mean ± s.e., n = 10 and n = 6 for naïve and 750 pulses, respectively (**B**, right graphs). *p < 0.05, ** p < 0.01 for the difference between 750 pulses and naïve groups.

Figure 1A (top panel) shows that both 500 and 750 pulse (200-ns, 25 kV/cm, 2 Hz) doses caused an initial shrinkage of the primary tumor to level unmeasurable by the caliper. Incompletely treated tumor started to relapse at 10 to 15 days post treatment in both experimental groups. However, 750 pulses were much more efficient with 75% (6/8) of the animals still tumor free at 4 weeks after nsPEF compared to 37.5% (3/8) for the 500 pulse group. (Fig. 1A, bottom panel).

In order to assess the antitumor immune response elicited by the pulse treatment, tumor-free mice in both experimental groups were challenged with B16F10 cells at 7 weeks post nsPEF. An age matched naïve group was used as a control for tumor growth. In the 500 pulse group all animals developed secondary tumors (3/3; Fig. 1B, left graphs). Conversely, in the 750 pulse group secondary tumors grew significantly slower than in naïve mice and 33% (2/6) of the animals remained tumor free for up to 50 days after challenge (Fig. 1B, right graphs).

Collectively, these results show that 200-ns pulses ablate primary B16F10 tumors in a dose-dependent manner and that the treatment induces an antitumor immune response.

### Histological analysis of nsPEF treated tumors revealed extensive damage not associated with caspase 3 activation or immune cell infiltration

**Figure 2:**
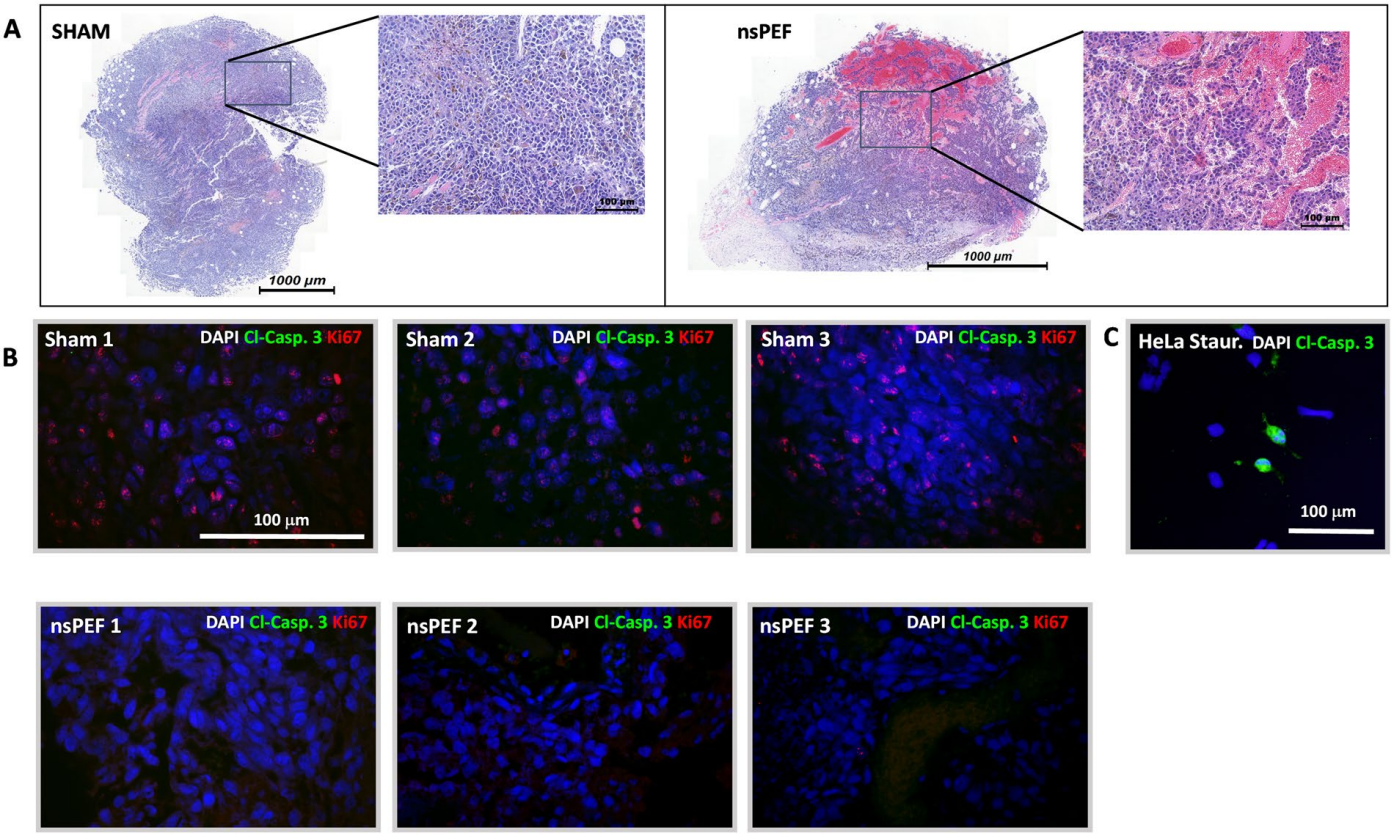
Histological analysis of nsPEF treated tumors. 30–50 mm^3^ B16F10 tumors were treated with 750, 200-ns pulses (25 kV/cm, 2 Hz) or left untreated (sham control). Panel A shows H&E pictures for one sham and one nsPEF-treated tumor collected at 4 h post treatment. In (**B**), both anti-cleaved caspase 3 (green) and -Ki-67 (red) immunofluorescence were performed to assess apoptosis and cell proliferation, respectively. Panel B shows representative images from three sham (top) and three nsPEF (bottom) -treated tumors. Panel C shows a positive control for the anti-cleaved Caspase 3 staining, namely HeLa cells treated with 1 μm staurosporin for 5 h. Scale bar: 1000 μm or 100 μm (inset) (**A**); 100 μm (**B**,**C**).

ICD depends on the activation of a multi-module signaling pathway that eventually results in the emission of DAMPs. Several studies reported that cells undergoing apoptosis can stimulate a tumor-specific immune response^4,51,52^. To study the effect of the treatment *in vivo*, we collected 3 nsPEF-treated tumors (750, 200-ns pulses, 25 kV/cm, 2 Hz) and 3 sham controls at 4 hours post treatment. Analysis of H&E-stained sections revealed extensive tissue damage in all nsPEF treated tumors (Fig. 2A) with no sign of immune cell infiltration. The damage caused by nsPEF was confirmed by the absence of active proliferating cells (Ki67 positive cells) in the treated tumors as compared to sham-exposed control samples (Fig. 2B). Cell death in nsPEF treated tumors was not accompanied by Caspase 3 activation suggesting that apoptosis was not activated in response to the treatment (Fig. 2B).

Altogether our results show that nsPEF cause rapid and extensive damage to B16F10 tumors which is not associated with caspase 3 activation.

### 200-ns pulses failed to trigger apoptosis in B16F10 cells

**Figure 3:**
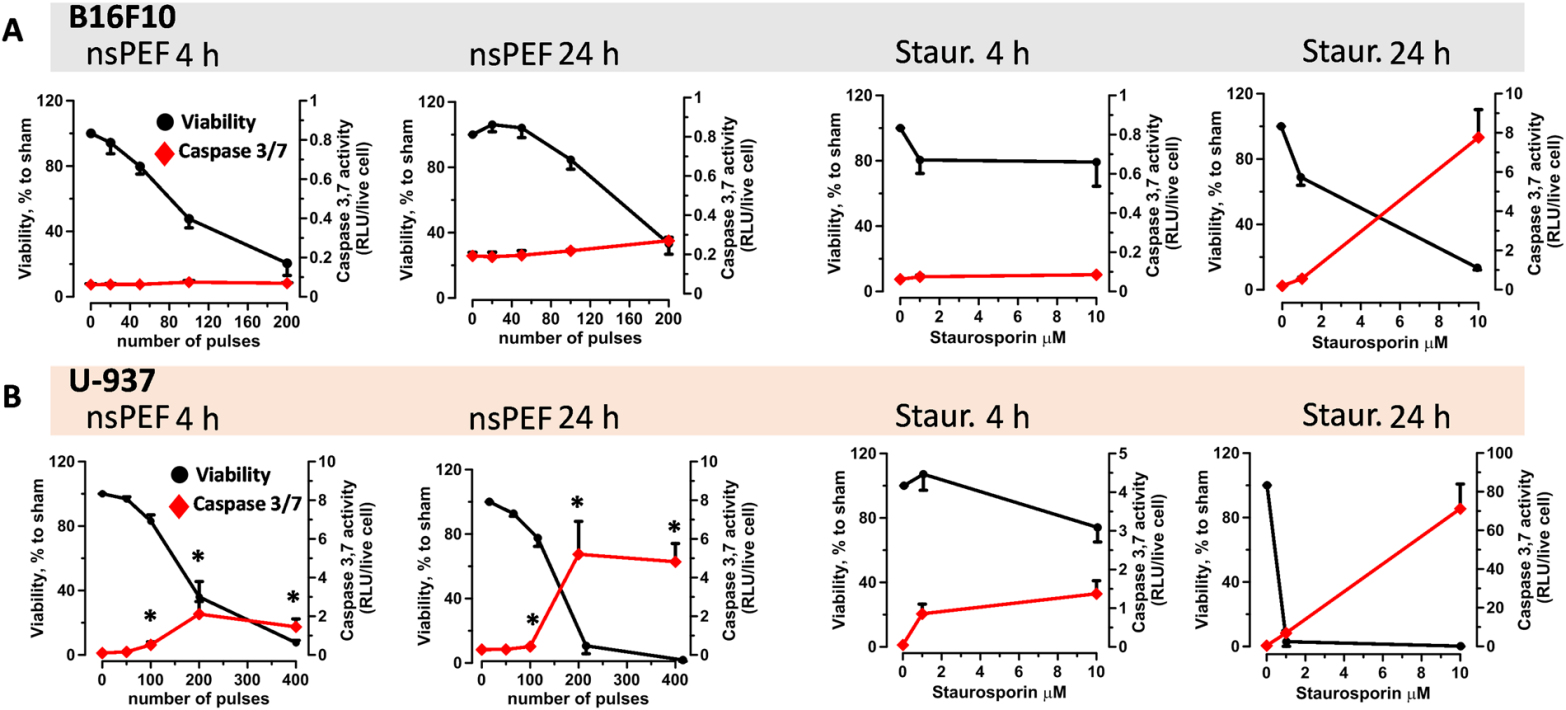
nsPEF triggers apoptotic cell death in U-937 but not in B16F10 cells. B16F10 (**A**) and U-937 (**B**) cells were either exposed in cuvettes to increasing numbers of 200-ns pulses (7 kV/cm, 10 Hz) or treated with staurosporine. Both cell viability (Presto blue assay) and Caspase 3/7 activation (Caspase-Glo 3/7 assay) were measured at 4 and 24 hours post treatment. In each plot the left y-axis refers to cell vibility expressed in %-to sham exposed parallel control (shown in black) while the right y-axis is the caspase activity expressed in relative luminescence units (RLU) per live cell (shown in red). Mean +/− s.e. n = 3–5. *p < 0.05 for caspase activity of nsPEF from sham.

Apoptosis induction in response to nsPEF has been documented in multiple cell lines including B16F10^53^. We therefore decided to further investigate apoptosis in B16F10 cells and to compare it with monocyte lymphoma U-937, a cell line were we previously reported apoptotic cell death in response to nsPEF^26,41^. Cells were exposed to increasing numbers of 200-ns pulses (7 kV/cm, 10 Hz) and both viability and caspase 3/7 activity were measured at 4 and 24 h post treatment (Fig. 3). The use of different number of pulses for B16F10 and U-937 reflects their different sensitivity to nsPEF, with B16F10 being more sensitive than U-937 cells. Fig. 3A shows that in B16F10 200-ns pulses did not induce caspase activation although the pulse treatment severely impaired cell viability. Conversely, in U-937 cells nsPEF increased caspase activation already at 4 h post nsPEF (Fig. 3B). Caspase 3/7 activation increased 2–6 fold at 24 h, correlating with a strong decrease in cell viability. Notably, U-937 were also remarkably more sensitive than B16F10 to Staurosporin induced apoptosis.

**Figure 4:**
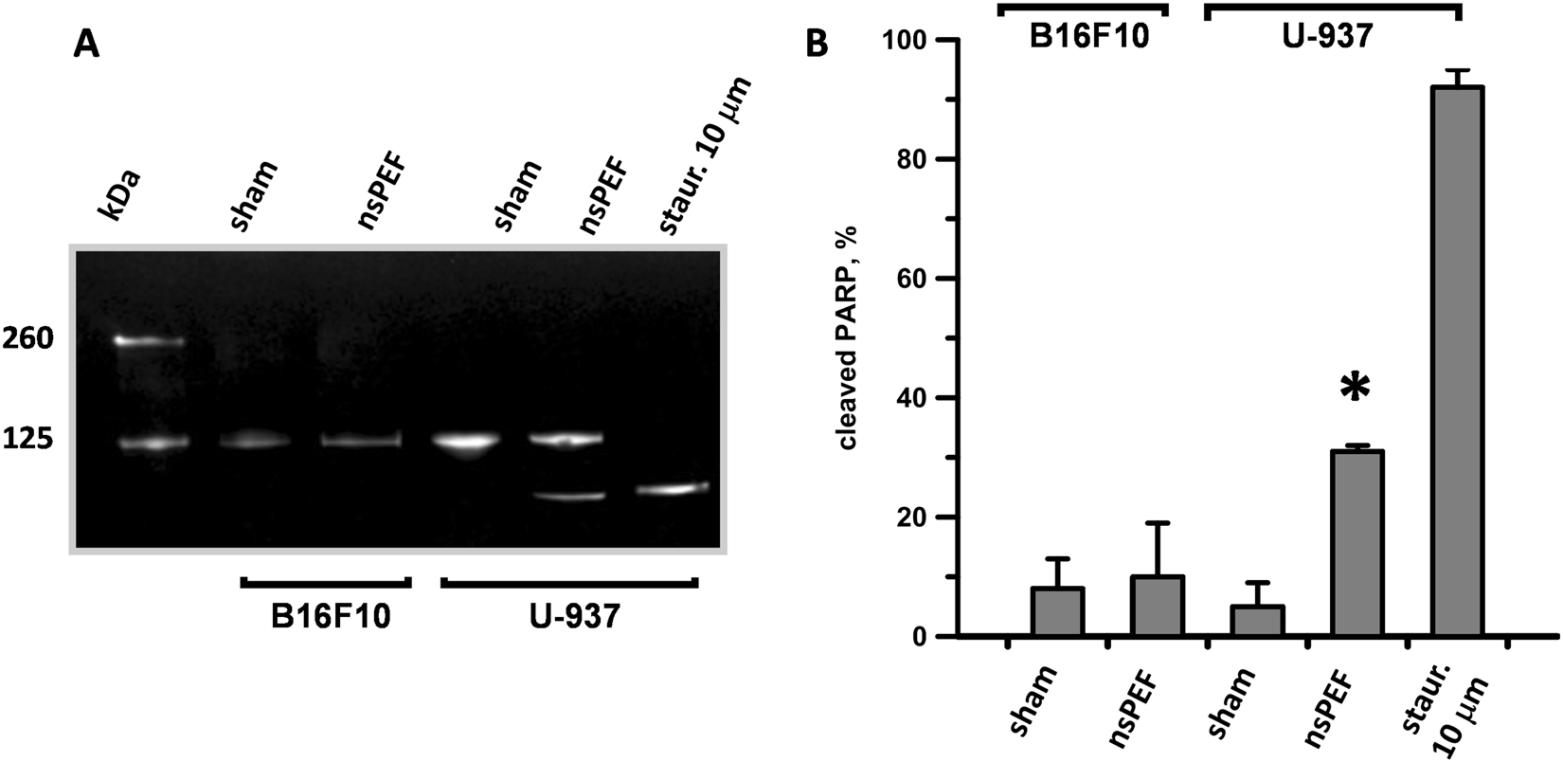
nsPEF induce PARP cleavage in U937 but not in B16F10. B16F10 and U-937 cell suspensions were exposed to 100 pulses (200-ns, 7 kV/cm, 10 Hz) or left untreated (sham). Protein extracts collected 6 hours after treatment were analyzed by western blot for full length (116 kDa) and cleaved PARP (89 kDa). Panel A shows a representative Western blot and (**B**) the quantification of the PARP cleaved fraction. Staurosporine (10 µM) treated U937 were used as a positive control. Blot image has been cropped, full-length blot is presented in Supplementary Figure 1. Mean +/− s.e. n = 3. *p < 0.01 for the difference between sham and nsPEF-treated U-937.

The lack of apoptosis in nsPEF-treated B16F10 was confirmed by using another hallmark of apoptosis, namely the quantification of PARP cleavage. While in U-937 30% of PARP was cleaved at 6 h after nsPEF, in B16F10 the pulse treatment had no effect (Fig. 4).

Overall we proved that 200-ns pulses failed to induce apoptosis in B16F10 suggesting that the ablative and protective effects seen *in vivo* are not dependent on this cell death pathway. Our results contradict a previous study reporting activation of apoptosis in B16F10 in response to nsPEF^53^. However, it should be noted that Ford *et al*. used much higher electric fields (12–60 kV/cm).

### B16F10 cells lack RIP3 expression and are resistant to necroptotic stimuli

**Figure 5:**
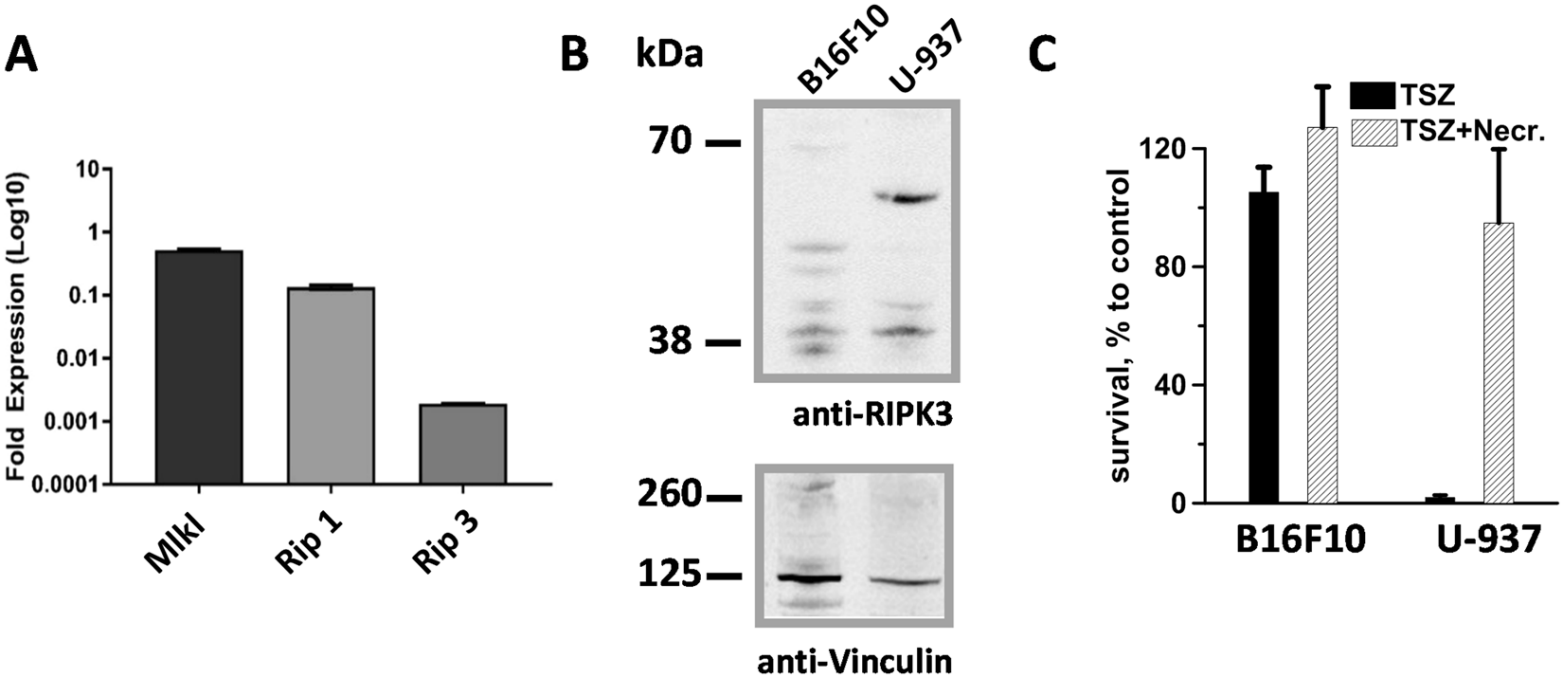
Necroptotic machinery expression analysis and response to necroptotic stimuli: B16F10 vs. U937 cells. In panel A the expression levels of Mlkl, Rip1 and Rip3 genes in B16F10 cells were measured by real-time quantitative PCR. Each gene mRNA level was normalized to the housekeeping *Hprt* gene mRNA and is shown as relative expression. (**B**) Protein extracts from B16F10 and U-937 were analyzed by western blot for RIP3 and, as control, Vinculin expression. RIP3 (57 kDa) is expressed in U-937 but not in B16F10 lysates. Blot images have been cropped, full-length blots are presented in Supplementary Figure 1. (**C**) B16F10 and U-937 cells were treated with TSZ (25 ng/ml TNF alpha, 1 µM Smac mimetic and 40 μM zVAD). To block necroptosis we used 60 μm Necrostatin. Cell survival was measured at 24 h post treatment by MTT assay. Mean +/− s.e. n = 3 (**A**,**C**).

Because cells undergoing necroptosis were shown to exhibit all biochemical features of ICD^5^, we studied this cell death pathway in B16F10 cells. We first investigated whether B16F10 express the necroptotic machinery, namely receptor interacting protein kinase-1 (RIP1) and −3 (RIP3) and the pseudokinase mixed lineage kinase domain-like (MLKL). Consistent with the literature^54^, we found that B16F10 cells lack RIP3 expression, as measured by RT-PCR (Fig. 5A) and western blot analysis (Fig. 5B), and are therefore completely resistant to necroptosis induction by combination of TNF-α, the pan-caspase inhibitor zVAD and the Smac mimetic BV6 (from now on referred as TSZ treatment) (Fig. 5C). Conversely, in U-937, which endogenously express RIP3, treatment with TSZ decreased cell viability. Notably, cells were rescued by the RIP1 inhibitor necrostatin 1 confirming the occurrence of *bona fide* necroptosis (Fig. 5C).

### nsPEF caused rapid cell death in B16F10 *in vitro*

The lack of activation of programmed cell death pathways suggested that nsPEF have triggered necrosis in B16F10 cells. Indeed, several studies reported necrosis due to the colloid-osmotic cell swelling as a predominant mechanism of cell death after exposure to nsPEF^26,45^.

**Figure 6:**
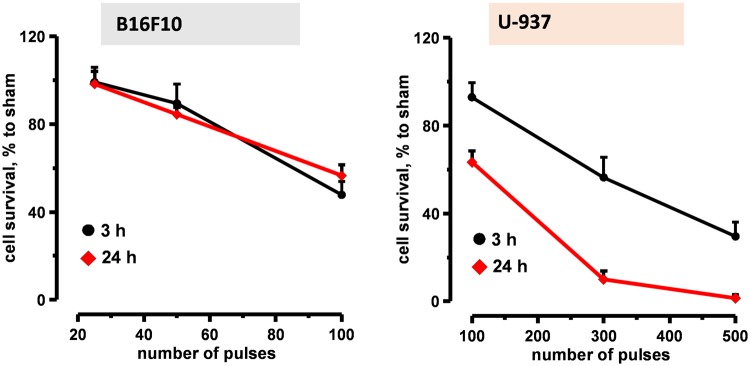
Time course of nsPEF induced cell death in both B16F10 and U-937 cells. Both B16F10 (left panel) and U-937 (right panel) were exposed to increasing number of 200-ns pulses (7 kV/cm, 10 Hz). Viability was measured at 3 and 24 h post treatment by Presto blue assay and expressed in %-to sham exposed parallel control. Mean +/− s.e. n = 5.

Figure 6 shows the time course of nsPEF-induced cell death in both B16F10 and U-937 cells. Cells were exposed to increasing number of 200-ns pulses (7 kV/cm, 10 Hz) and cell viability was measured at early (3 h) and late (24 h) time points (Fig. 6). At 3 h post exposure, 50 and 100 pulses killed 11 and 53% B16F10 cells, respectively. At 24 h we did not observe a further increase in cell death suggesting that the majority of cell death occurred early after electroporation. In U-937, at 3 h post nsPEF, 100 and 300 pulses caused 8 and 38% cell death, respectively. However over time cells kept dying and at 24 h post treatment 100 and 300 pulses killed 38 and 91.3% of the cells, respectively. These results support the activation of slower programmed cell death pathways in nsPEF-treated U-937.

Taking into account the early occurrence of the cell death, and the lack of concurrent caspase 3/7 activation or PARP cleavage, the cell death in B16F10 can be categorized as a primary necrosis.

### Autophagy is not induced in nsPEF-treated B16F10 cells

Autophagy, a catabolic process evolved to maintain the homeostasis of cellular components, has been shown to regulate ATP release during ICD^55^. Because nsPEF were found to activate autophagy^25^ we investigated whether this stress response was induced in response to 200-ns in B16F10 cells.

**Figure 7:**
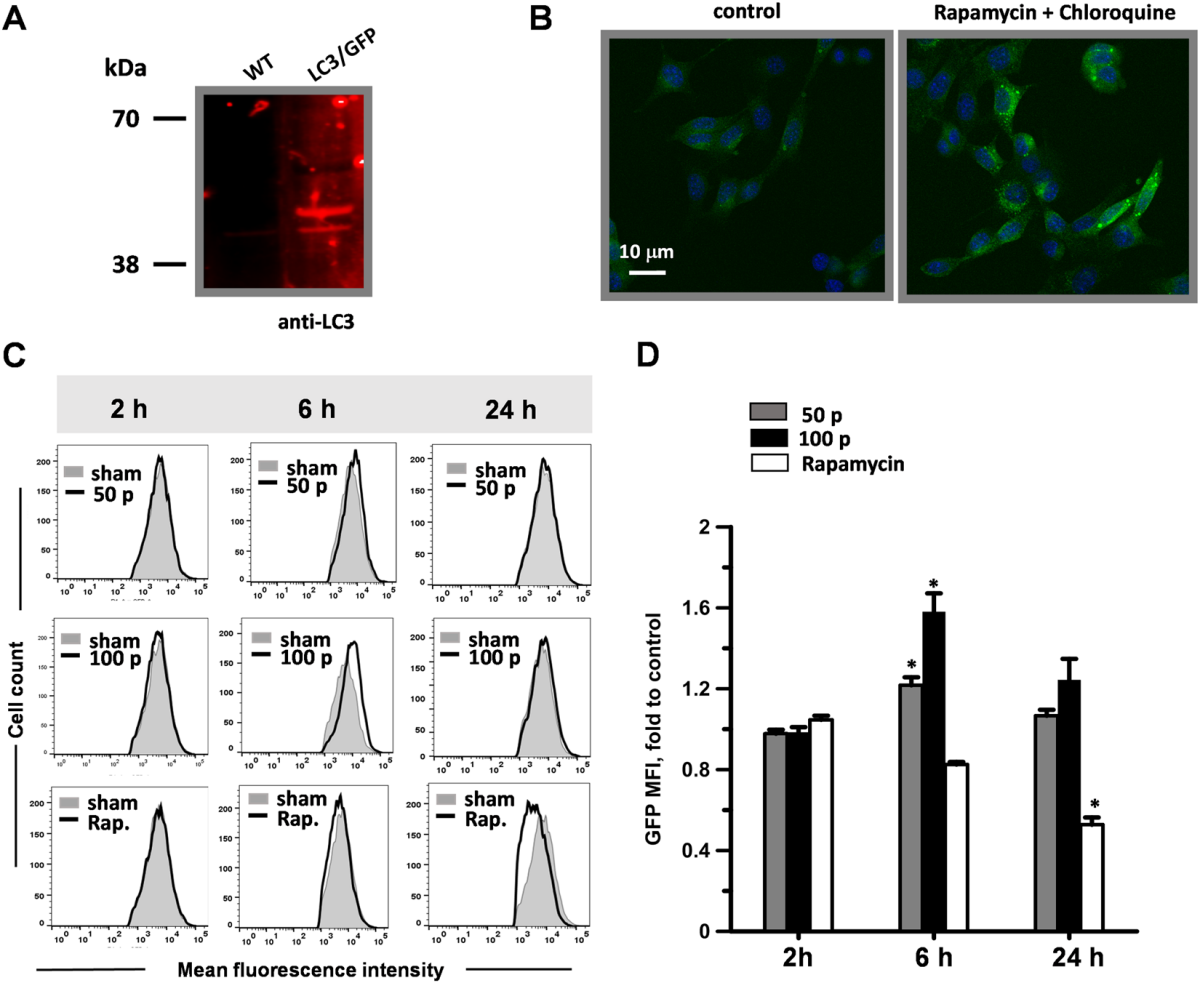
Effect of 200-ns pulses on B16F10 autophagy. A LC3/GFP stable B16F10 cell line was generated by transient transfection with a LC3/GFP construct and selection with G418. In (**A)** protein extracts from B16F10 and LC3/GFP B16F10 cells were analyzed by western blot for LC3 expression. LC3-GFP fusion protein is seen as a 50 kDa band. Blot image has been cropped, full-length blot is presented in Supplementary Figure 1. Panel B shows representative images from LC3/GFP B16F10 (control) and cells treated for 4 h with 1 μg/ml Rapamycin to induce autophagy and 25 μm Chloroquine to block LC3-GFP lysosome degradation. Scale bar: 10 μm. In (**C**) LC3/GFP B16F10 cells were exposed to either 50 or 100, 200-ns pulses (7 kV/cm, 10 Hz). As a positive control for autophagy induction cells were treated with rapamycin (1 μg/ml). The GFP mean fluorescence intensity was measured by flow cytometry at 2, 6 and 24 hours after treatment. Sham-exposed negative controls are shown in grey in all panels. (**D**) Shows the quantification of the effect seen in (**C**) Mean +/− s.e. for n = 3. *p < 0.01 for the effect of 50, 100 pulses and rapamycin vs. sham control.

During autophagy, the cytoplasmic LC3 is processed and recruited to the autophagosomes. The hallmark of autophagic activation is thus the formation of cellular autophagosome puncta containing LC3. In order to study autophagy, we generated a B16F10 cell line stably expressing LC3 fused to GFP (LC3/GFP B16F10; Fig. 7A). In preliminary experiments we investigated how the LC3/GFP B16F10 cell line responded to autophagic stimuli. Cells were treated for 4 h with the autophagy inducer Rapamycin (1 μg/ml) and Chloroquine (25 μM). Chloroquine was added because it inhibits the fusion of autophagosomes with lysosomes making it easier to detect the LC3 puncta by fluorescence microscopy. Fig. 7B shows that autophagy stimuli induced the formation of LC3/GFP puncta proving that this cell line was a suitable tool to investigate autophagy.

To measure the autophagic activity in response to nsPEF, we took advantage of a new method which uses flow cytometry to quantify the turnover of GFP/LC3^56^. GFP/LC3 is specifically delivered into lysosomes in response to autophagy induction and therefore the fluorescence intensity of GFP/LC3 is reduced in a time-dependent manner. We treated LC3/GFP B16F10 with 50 and 100, 200-ns pulses (7 kV/cm, 10 Hz) and with rapamycin as a positive control. GFP mean fluorescence intensity was measured at 2, 6 and 24 h post treatments. Fig. 7C shows that at 24 h post treatment rapamycin caused a decrease in LC3/GFP mean fluorescence intensity suggesting the activation of the autophagic flux in the cells. Conversely, both nsPEF doses caused a statistically significant increase in LC3/GFP fluorescence at 6 h post treatment suggesting a block rather than induction of the endogenous autophagy (Fig. 7D). However, this block was transient and at 24 h post treatment was no longer detectable.

### nsPEF and surgery yield similar antitumor immunity

**Figure 8:**
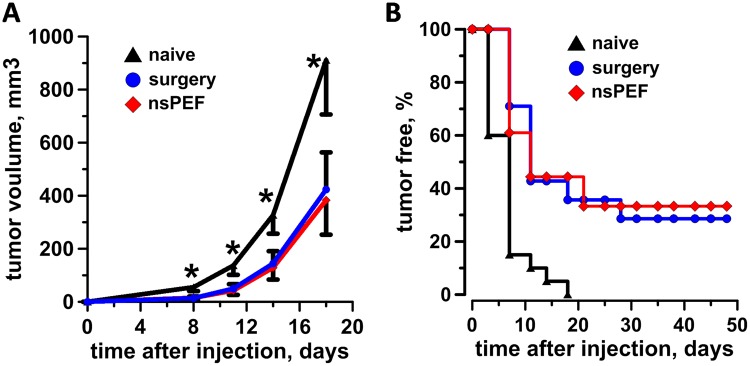
Antitumor immune response in nsPEF vs. surgery treated mice. 40–50 mm^3^ B16F10 tumors were either treated with nsPEF (750, 200-ns pulses, 25 kV/cm at 2 Hz) or surgically removed. At 7 weeks after treatments, animals that experienced complete tumor remission were challenged with tumor cells and monitored for the appearance of palpable tumors. An age matched naïve group was used as a control for tumor growth. Panel A shows the tumor growth curves and (**B**) the % of tumor free animals. Mean +/− s.e., n = 20, 18, and 14 for naïve, nsPEF and surgery groups, respectively. *p < 0.05 for the difference of nsPEF from naïve.

Because nsPEF failed to trigger the main pathways involved in ICD, we asked whether the local ablation by nsPEF improved the anti-B16F10 tumor immunity that naturally arises in immunocompetent C57BL/6 mice. This basal antitumor immunity stays dormant co-existing with the progressing tumor but can be restored when the tumor is removed by surgery^57^. To address this point, B16F10 tumors were either treated with nsPEF (750, 200-ns pulses, 25 kV/cm, 2 Hz) or surgically removed when they reached on average size of 40–50 mm^3^ (Table 1). The treatment with nsPEF was slightly more efficient at curing animals with 15% of tumor relapse compared to 18.7% in the resected surgery group. At 7 weeks post treatments, tumor-free animals in both experimental groups were challenged with a secondary tumor. Figure 8A shows that the two treatments equally impaired the growth of secondary tumors. Moreover, no statistically significant differences in tumor free survival were observed, nsPEF protected 33% (6/18) of the animals and surgery 28.6% (4/14) (Fig. 8B). These results suggest that nsPEF reinstated but did not enhance the natural occurring antitumor immunity.

**Table 1:** Average size of the tumors at time of nsPEF treatment or surgery and % of tumor relapse in both experimental groups.

groups	nsPEF	surgery
Average tumor size at time of treatment, mm^3^	48.6 +/− 2.3	45.2 +/− 3
Tumor relapse %	15	18.7

## Discussion

NsPEF can efficiently ablate tumors *in vivo* but the capacity to initiate an effective antitumor immunity is variable. While some studies showed that nsPEF induced 100% protection against tumor challenge^8,9^, other groups reported a long-lasting antitumor immunity in only 25% of the treated animals^10^. These studies used rather different electric pulse parameters and tumor cell models, suggesting that these factors may affect and/or drive the immunogenicity of nsPEF treatments.

In this study we correlated the protective antitumor response induced by 200-ns pulses in B16F10 syngeneic mice with the cell death and stress modalities triggered by the pulse treatment. Our results show that nsPEF caused primarily necrosis. B16F10 cell death occurs within the first 3 h post nsPEF with no sign of concurrent caspase 3/7 activation or PARP cleavage.

Necrosis is a forced accidental type of cell death characterized by the loss of the cell membrane integrity, release of intracellular content and associated inflammation. Despite these immunogenic characteristics, *in vivo* necrotic cell vaccines have shown an immunological inert nature and although local inflammation and infiltration of immune cells at the injection site occurred, it did not generate a productive adaptive immune response and did not protect animals from tumor development^2,3^. The immune response depends on the molecules presented or released by dying cells. Primary necrosis is a sudden event which causes the accidental release of almost unmodified DAMPs. In cells undergoing apoptosis, in contrast, caspases cause multiple molecular rearrangements that modify the inflammatory activity of DAMPs, which are then released during secondary necrosis. In addition to which DAMPs are released by the dying cell, the timing of such a release is critical. For instance Maueröder *et al*. recently reported that cell death induced by the tuberculosis-necrotizing toxin (TNT) was associated with a poor immune response as compared to cells overexpressing a constitutive active form of caspase 3. The author proposed that the poor immunogenicity of TNT overexpressing cells was caused by a fast release of “find me” signals like ATP in the temporal absence of DAMPs like heat shock protein 90 (HSP90) and HMGB1^58^.

With the lack of activation of cell death pathways involved in the active emission of DAMPs, it should come as no surprise that in B16F10 melanoma nsPEF induce a weak antitumor immune response. Indeed, nsPEF protected 33% of the animals contrary to 100% reported for both rat hepatocellular carcinoma^8^ and mouse mammary carcinoma^9^. Moreover, the percentage of protected animals was similar to the parallel surgery group where tumors were resected instead of being treated with nsPEF. Several studies have established that the antitumor immunity can be reinstated when the tumor is removed by surgery^59–61^. To explain this phenomenon it has been proposed that the complete removal of tumor burden and, therefore, the clearance of tumor antigens, favors the formation of an immune memory response^57^. Indeed, it has been shown that antigen persistence prevents memory formation and promotes immune exhaustion or tolerance^62,63^. In addition, to allow the formation of a memory response, surgery removes the antitumor immunosuppressive source. Several studies have assessed immunosuppression after tumor surgery and most found at least partial recovery of immune function following treatment^59,60,64,65^. Considering our data that nsPEF and surgery yield the same protection, one can speculate that nsPEF unleash the natural occurring antitumor immunity by offsetting the tumor immunosuppressive milieu.

To date, two studies reported that nsPEF induced an effective antitumor immunity which protected all treated animals from tumor challenge^8,9^. These studies used different orthotopic tumor models, namely the rat N1-S1 hepatocellular carcinoma^8^ and the mouse 4T1 mammary carcinoma^9^. In these studies, similarly to our experiments, tumors were allowed to grow for 7–10 days before nsPEF and, 7 weeks after treatment, tumor free animals were challenged with fresh tumor cells. Correlating with the high tumor protection efficiency, Chen *et al*. reported activation of the effector caspases 3 and 9 while Guo *et al*. measured an increased release of DAMPs (CRT, ATP and HMGB1) in nsPEF-treated 4T1 cells.

In light of these results a key question is why nsPEF failed to induce ICD in melanoma cells.

Comparing our pulse treatment (750 200-ns pulses, 25 kV/cm, 2 Hz) with both Chen *et al*.^8^. and Guo *et al*.^9^. (1000 100-ns pulses, 50 kV/cm, 1–2 Hz), we used pulses which are twice longer at lower doses. A central role for ER stress has been revealed in all scenarios of ICD described thus far^6^. NsPEF could trigger ER stress through at least two mechanisms. First, by directly permeabilizing the ER leading to a rise in cytosolic Ca^2+ ^^17^^,^^21^^,^^66^. The acute release of Ca^2+^ from the ER can severely impact protein folding capacity and trigger Ca^2+^-mediated mitochondrial cell death^67^. Second, by inducing reactive oxygen species (ROS) production^29,30^. Notably, most ICD stimuli including anthracyclines, UVC radiation and hypericin-photodynamic therapy (Hyp-PDT) trigger ROS-dependent ER stress. With the use of nanosecond pulses, as pulses shorten, more intracellular effects and less effects on plasma membrane can be expected^18,68,69^. If the pulse width is shorter than the charging time of the plasma membrane the electric field will pass through the membrane into the cytoplasm and affect internal cell structures^68^. If the amplitude of the applied field is high enough, transmembrane voltages across intracellular membranes will reach threshold values, and pore formation in such membranes becomes likely. Because characteristic charging time constants for the plasma membrane of mammalian cells are on the order of 100-ns^68^ one can speculate that 1000, 100-ns pulses at 50 kV/cm (as used in both Chen *et al*. and Guo *et al*.) have compromised the ER homeostasis of both hepatocellular and breast carcinoma more than 750, 200-ns pulses at 25 kV/cm in melanoma tumors. Indeed, Nuccitelli *et al*. recently reported that 100-ns pulses triggered apoptotic cell death accompanied by CTR exposure, ATP and HMGB1 release in three different tumor cell types (MCA 205 murine fibrosarcoma, Jurkat E6-1 human T-cell leukemia and McA-RH777 rat hepatocarcinoma)^10^. Whether shorter pulses induce more ER-stress and therefore DAMPs emission has yet to be explored.

Besides the pulse treatment, also the tumor model may affect the immunogenicity of nsPEF treatment. For instance, we found that B16F10 cells were remarkably more resistant than U-937 to Staurosporin-induced apoptosis. Moreover, we reported that B16F10 cells do not express RIP3 and, therefore, are completely resistant to necroptotic stimuli. These findings suggest that tumor cells which harbor genetic or epigenetic alterations that compromise the cell death signaling pathway may also inhibit ICD.

Taken together, we showed that 200-ns pulses triggered necrosis and efficiently ablated melanoma tumors unleashing, but not boosting, the natural occurring antitumor immunity. Further research will be focused on gaining a better understanding of how pulse parameters affect DAMPs emission in order to enhance the immunogenicity of nsPEF-induced cell death.

## Materials and Methods

### Cell culture and stable cell lines

Mouse melanoma B16F10 (ATCC, Manassas, Virginia) and human cervix carcinoma HeLa cell lines were cultured in Dulbecco’s Modified Eagle Medium (DMEM, Corning®, Corning, NY) while the human lymphoma U-937 cell line was cultured in RPMI 1640 (Corning®). Culture media were supplemented with L-glutamine (ATCC), 10% fetal bovine serum (Atlanta Biologicals, Norcross, GA), 100 U/ml penicillin and 0.1 mg/ml streptomycin (Gibco, Gaithersburgh, MD).

For B16F10 overexpressing the microtubule-associated protein light chain 3 (LC3) fused to GFP (GFP/LC3-B16F10), cells were transfected with pEGFP-LC3 plasmid (Addgene, Cambridge, MA) using Lipofectamine 2000 (ThermoFisher Scientific, Waltham, MA) according to the manufacturer’s instructions. Cells were then selected with 2 mg/ml of geneticin (G418, Sigma-Aldrich, Saint Louis, MO) and single clones were analyzed for expression of both LC3 and GFP.

### Pulsed electric field exposure methods

In the *in vitro* experiments, cell samples were exposed to nsPEF in 1 mm gap electroporation cuvettes (BioSmith, San Diego, CA) at room temperature (22 ± 2 °C). Cells were resuspended at 1.2 to 2 × 10^6^ cell/ml and 100 μl samples of this suspension were loaded in the electroporation cuvettes and subjected to either nsPEF or sham exposure. Trains of trapezoidal 200-ns pulses were delivered to cuvettes from an AVTECH AVOZ-D2-B-ODA generator (AVTECH Electrosystems, Ottawa, Ontario, Canada) via a 50- to 10-Ohm transition module (AVOZ-D2-T, AVTECH Electrosystems) modified into a cuvette holder. Samples were exposed to up to 500, 200-ns pulses, 7 kV/cm at 10 Hz. Maximum adiabatic heating from nsPEF did not exceed 7 °C, as calculated by adiabatic heat equation. Once exposure was completed, cells were seeded in triplicate in either 96 or 24 well plates and incubated at 37 ° C in the incubator for the different incubation times (2 to 24 h).

**Figure 9:**
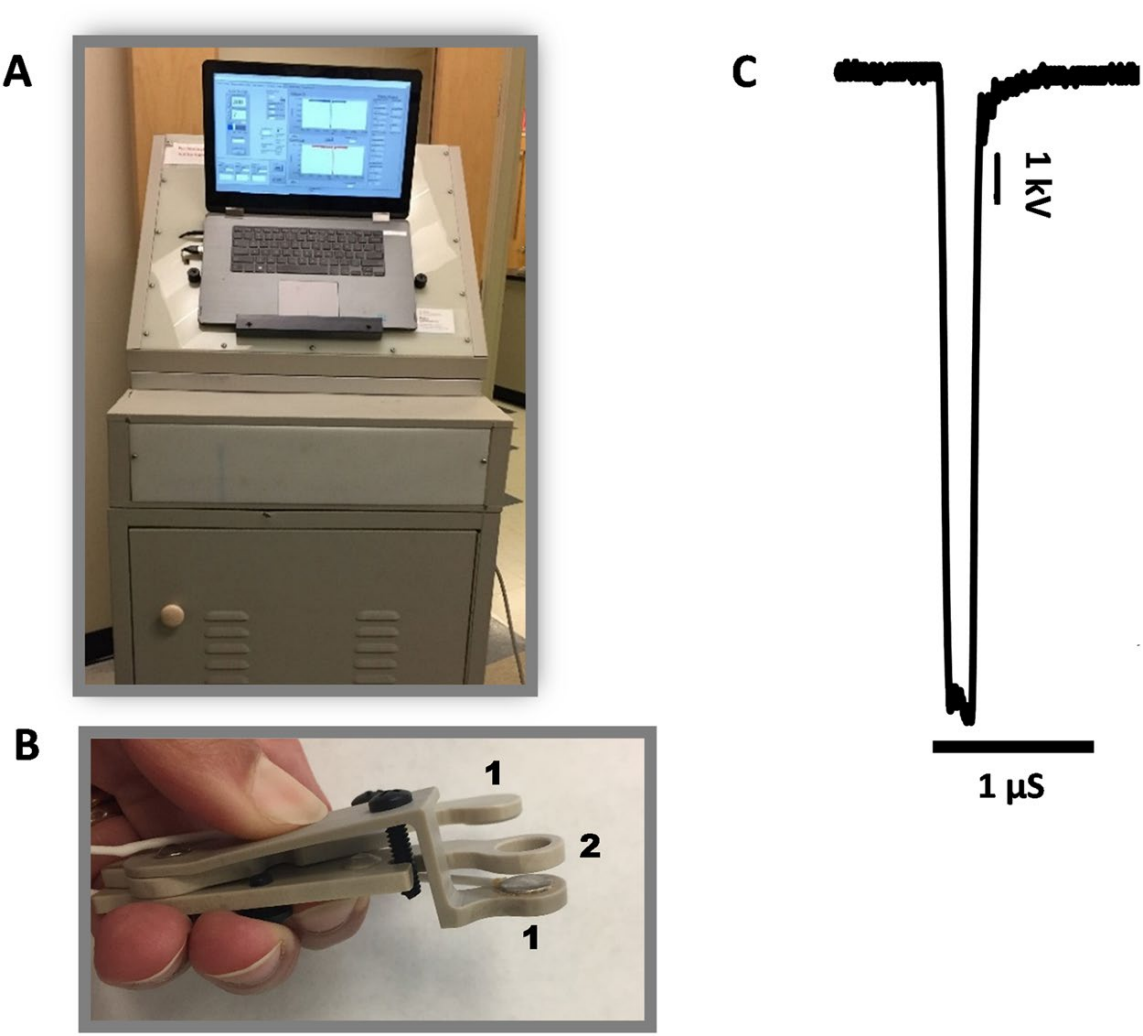
nsPEF exposure setup to treat mice tumors. (**A**) Pulse generator. (**B**) Plate pinch electrode with two round stainless steel plates (1) 8 mm in diameter and a spacer of 4 mm between the plates (2). (**C**) Representative waveform (200 ns) at 10 kV.

To treat B16F10 tumors *in vivo*, mice were anesthetized by inhalation of 3% isoflurane in oxygen (Patterson Veterinary, Devens, MA). Trapezoidal pulses of 200-ns duration were produced by a custom pulse generation system with an output impedance of 100 Ω, adjustable pulse amplitude (up to 15 kV), duration (200 to 1000 ns) and frequency (1–100 Hz; Pulse Biosciences, Inc., Hayward, CA; Fig. 9A). Pulses of 200-ns duration (500–750, 2 Hz, 25 kV/cm) were applied using an electrode that sandwiched the tumor between two flat round polished stainless-steel plates with a spacing of 4 mm between the two plates (Fig. 9B). The electric field was calculated as: E = V/d where V is the applied voltage and d is the distance between the two plate electrodes. Ultrasound conductive gel (Parker Laboratories, Fairfield, NJ) was used to ensure an efficient electrical continuity. The waveform of a 200 ns pulse at 10 kV is reported in Fig. 9C. Animals in the sham control group underwent anesthesia and the probe insertion procedure but no nsPEF delivery.

### Viability and caspase 3/7 activity assays

Viability was measured at 4 and 24 hours after nsPEF treatment using the resazurin-based metabolic assay Presto Blue (Life Technologies, Grand Island, NY). Briefly, 5 µl of Presto Blue reagent were added to each well for 1 hour at 37 °C in the incubator. Plates were read with the Synergy 2 microplate reader, with excitation/emission settings at 530/590 nm.

In certain experiments, concurrently with cell viability, caspase 3/7 activity was measured. We first recorded fluorescence (Presto Blue/viability) and then added the Caspase -Glo 3/7 assay (Promega Corporation, Madison, WI) according to manufacturer’s instructions. As a positive control for apoptosis induction, cells were treated 1–10 μm staurosporine.

Cell viability after necroptosis induction using TNF-α (25 ng/mL; ApexBio, Huston, TX), the pan-caspase inhibitor zVAD (40 µM; ApexBio, Huston, TX) and the Smac mimetic BV6 (1 µM; ApexBio, Huston, TX), was assessed by MTT (BioAssay Systems, Hayward, CA) assay according to the manufacturer’s instructions. In brief, 10 µl of the MTT reagent were added to 100 µl of cell suspension and incubated for 2 h until adding the solubilization buffer (10% SDS, 0.01% HCl in water). The plates were left on an orbital shaker for 2 h and the absorbance was read at 570 nm.

Samples were measured in triplicates, the data were averaged, corrected for the background, and considered as a single experiment.

### Murine tumor model and surgical procedure

Seven- to 8-week-old C57/BL6 female mice (Jackson Laboratory, Bar Harbor, ME) were housed in individually ventilated cages in groups of 5 under pathogen-free conditions. Animals were inoculated via intradermal injection in the right dorsolateral flank with 5 × 10^5^ B16F10 cells in 50 µl of PBS. Tumors were allowed to grow to a volume of approximately 40–50 mm^3^ before PEF treatment.

For primary tumor resection experiments, mice were anesthetized by inhalation of 3% isoflurane in oxygen, tumors of about 40–50 mm^3^ were removed and the incision closed using skin staples. To relieve pain after surgery, mice were injected subcutaneously with analgesics (Carprofen 4 mg/kg) immediately after treatment and for 3 days after surgery. Skin staples were removed at 10–14 days after treatment.

To measure the antitumor immunity, 7 weeks post nsPEF or surgery tumor free survivors were injected in the left dorsolateral flank with 5 × 10^5^ B16F10 cells in 50 µl PBS. An age-matched naïve group of mice was used as control for tumor growth. Animals were monitored for up to 50 days post challenge.

Tumor growth was measured at 24 h post treatment and twice weekly using a digital caliper, and volumes (*v*) were calculated using the standard formula *v* = *ab*^2^*π/6*, where *a* is the longest diameter, and *b* is the next longest diameter perpendicular to *a*. Mice were humanely euthanized when the tumor reached 800 mm^3^.

All procedures were carried out in accordance with the Guide for the Care and Use of Laboratory Animals, Eighth Edition and was approved by the Old Dominion University Institutional Animal Care and Use Committee (permit number: 17–012).

### Immunohistochemistry and immunocytochemistry fluorescent staining

nsPEF treated and sham controls tumor samples were fixed in 4% paraformaldehyde at 4 h post treatment and then shipped to IDEXX Labarotories, Inc. (Westbrook, ME) for paraffin embedding, sectioning, glass slide mounting and hematoxylin and eosin staining (H&E). Rehydration was accomplished using standard protocols, briefly, two washes in xylene, and subsequent washes in gradient alcohol (100, 95, 75, 50, and 25%), followed by rehydration in RO water. Samples were then boiled for 10 min in 10 mM citric acid (AR6, PerkinElmer, Waltham, MA) to expose epitopes. Cell permeabilization was performed for 20 min in 0.25% Triton X-100 in PBS. Blocking was performed with 4% bovine serum albumin (BSA) in PBS with 0.01% Tween 20 (PBST) for 1 h at room temperature. Samples were then incubated overnight at 4 °C with the primary antibodies. Primary antibodies were polyclonal rabbit anti-cleaved caspase 3 (Asp175) (Cell Signaling Technology, Danvers, MA) and mouse anti-Ki67 (eBioscience, San Diego, CA). After incubation with primary antibodies, samples were washed 5 times for 15 min with PBST, and incubated with corresponding secondary antibodies, goat α-rabbit IgG (H + L) AlexaFluor 488 (ThermoFisher Scientific) and goat α-mouse IgG (H + L) AlexaFluor 546 (Life Technologies, Carlsbad, CA) for one hour at room temperature. All antibodies were diluted 1:200 in blocking buffer. Samples were then counterstained with DAPI, mounted with VECTASHIELD® HardSet™ mounting medium (Vector Laboratories, Burlingame, CA) and allowed to set for 15 min at room temperature. The samples were then stored in −20 °C until imaging.

As a positive control for caspase 3 activation, HeLa cells attached to poly-L-lysine coated glass coverslips were treated with 1 μM staurosporine. At 4.5 h post treatment, cells were fixed in 4% paraformaldehyde in PBS for 20 min and then permeabilized for 4 min in 0.1% Triton X-100 in PBS. Blocking was performed with 4% BSA in PBS for 30 min at room temperature. Both primary anti- cleaved caspase 3 (Asp175) and secondary goat α-rabbit IgG (H + L) antibodies were diluted 1:200 in blocking buffer and incubated for 30 minutes at room temperature. Samples were mounted with Fluoroshield Mounting Media with DAPI (Abcam, Cambridge, UK) and stored at +4° C until imaging.

Samples were imaged using Olympus IX81 microscope equipped with an FV1000 confocal laser scanning system (Olympus America, Center Valley, PA). Fluorescence was quantified with MetaMorph Advanced v. 7.7.10.0 (Molecular Devices, Foster City, CA).

### Flow cytometry, RT-PCR and Western blot

At 2,6 and 24 h post nsPEF, B16F10 cells stably expressing GFP-LC3 were resuspended in PBS and GFP mean fluorescence intensity was measured by flow cytometry. Dead cells staining positive for propidium iodide (PI) were excluded from the analysis. Rapamycin 1 µg/ml (APExBIO) was used as a positive control for autophagy induction. Samples were acquired using a MACSQuant Analyzer 10 flow cytometer (Miltenyi Biotec, Bergisch Gladbach, Germany) and analyzed with FlowJo software (FlowJo, Ashland, OR).

The RT-PCR procedure was described in detail previously^70^. Gene expression analysis was conducted on samples using TaqMan Gene Expression Assays for *Rip1* (Mm00436354_m1), *Rip3* (Mm00444947_m1), *Mlkl* (Mm01244222_m1) and the endogenous housekeeping gene *Hprt* (Mm03024075_m1). Relative fold-changes were calculated using the 2^−ΔΔ*Ct*^ method. Significance was determined by comparing the 2^−Δ*Ct*^ value using a one-way analysis of variance with a Dunnett’s post hoc. Error bars represent the standard deviation of the relative-fold expression between samples.

The Western blot procedure was described in detail previously^26^. Membranes were blocked in Odyssey blocking buffer for 1 h at room temperature (LI-COR Biosciences, Lincoln, NE). Both primary antibodies, rabbit anti-LC3B and anti-RIP3, were from Cell Signaling while the rabbit anti-PARP polyclonal antibody was from Roche (Roche Diagnostics GmbH, Mannheim, Germany). The secondary donkey anti-rabbit IgG (H + L) antibody conjugated with an infrared fluorophore IRDye-680LT was from LI-COR Biosciences. Antibodies were diluted in 0.2% Tween-20 in Odyssey blocking buffer according to the manufacturer’s instructions. The membranes were incubated overnight at +4° C and for 1 h at room temperature with primary and secondary antibodies, respectively.

Membranes were imaged using Odyssey 9120 Infrared Imaging System (LI-COR Biosciences) in the 700 nm channel.

The fraction of the cleaved PARP (*K*, %) was calculated as: *K* = 100 × 1.3 *S*/(1.3 *S* + *L*) where *L* and *S* are the fluorescence intensities of the 116 kDa full-length PARP and of the 89 kDa PARP fragment, respectively. The coefficient 1.3 was used for *S* mass correction.

### Statistical analysis

Data are presented as mean ± standard error for *n* independent experiments. Statistical analyses were performed using a two-tailed *t* test where *p* < 0.05 was considered statistically significant. Statistical calculations, including data fits, and data plotting were accomplished using Grapher 11 (Golden Software, Golden, Colorado).

## Electronic supplementary material

Supplementary figure 1

## Data Availability

All data generated or analyzed during this study are available from the corresponding author on reasonable request.
