# Supplementary material for: Mechanisms and immunogenicity of nsPEF-induced cell death in B16F10 melanoma tumors
Source: Sci Rep. 2019 Jan 23;9:431. doi: 10.1038/s41598-018-36527-5 (PMC6344591; doi:10.1038/s41598-018-36527-5)

Mechanisms and immunogenicity of nsPEF-induced cell death in B16F10 melanoma tumors

Alessandra Rossi[1], Olga N. Pakhomova[1], Andrei G. Pakhomov[1], Samantha Weygandt[1], Anna A. Bulysheva[1], Len E. Murray[2], Peter A. Mollica[3], and Claudia Muratori[1*]

[1]Old Dominion University, Frank Reidy Research Center for Bioelectrics, Norfolk, VA 23508, USA

[2]SoBran Inc., 700 West Olney Road, Norfolk, VA 23507, USA

[3]Department of Medical Diagnostics and Translational Sciences, Old Dominion University, Norfolk, VA 23508, USA

* Claudia Muratori, PhD

Frank Reidy Research Center for Bioelectrics,

Old Dominion University

4211 Monarch Way, Suite 318

Norfolk, VA 23508

757 683 7044, 757 358 5889 (cell)

cmurator@odu.edu

Supplementary figure 1. A. Full-length blot for western blot shown in figure 4. **B**. Full-length blots for western blots shown in figure 5. **C.** Full-length blot for western blot shown in figure 7.

A

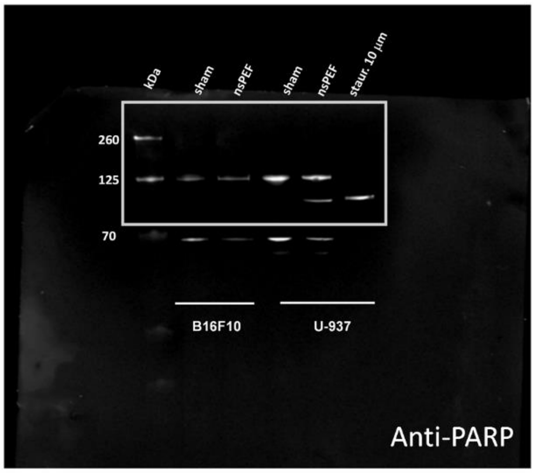

C

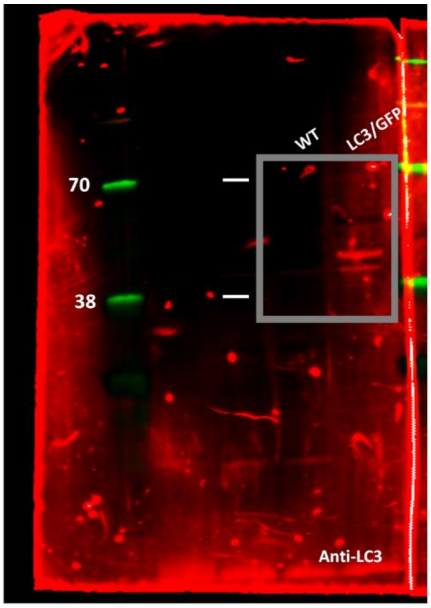

B

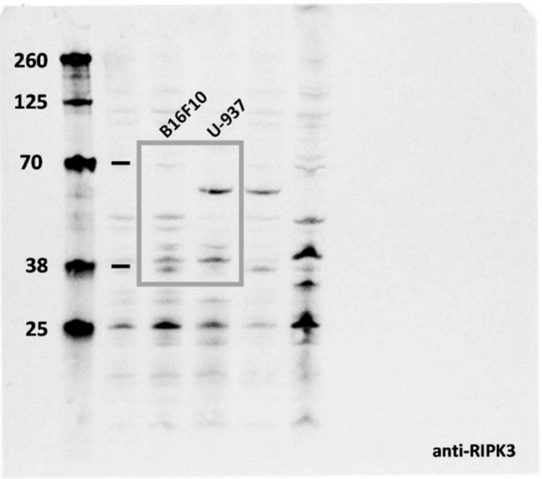

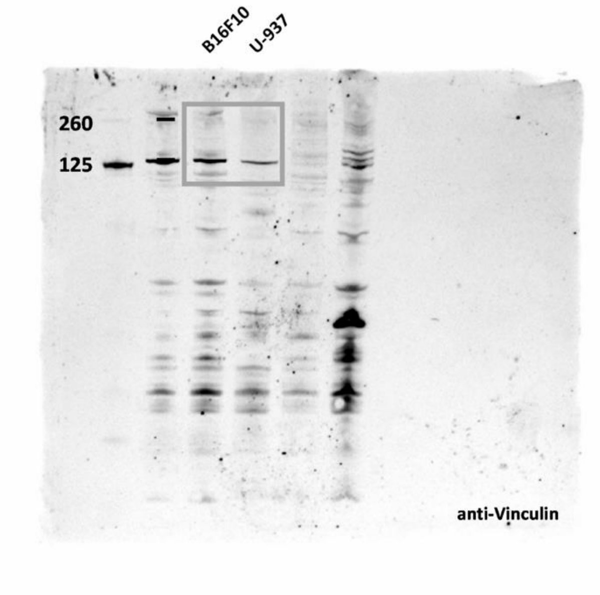

Supplement: Supplementary file 1 — Supplementary figure 1 [file 41598_2018_36527_MOESM1_ESM.pdf]
